# Evaluation of the Effectiveness of Charcoal, *Lactobacillus rhamnosus*, and *Saccharomyces cerevisiae* as Aflatoxin Adsorbents in Chocolate

**DOI:** 10.3390/toxins15010021

**Published:** 2022-12-28

**Authors:** Gamal M. Hamad, Amr Amer, Baher El-Nogoumy, Mohamed Ibrahim, Sabria Hassan, Shahida Anusha Siddiqui, Ahmed M. EL-Gazzar, Eman Khalifa, Sabrien A. Omar, Sarah Abd-Elmohsen Abou-Alella, Salam A. Ibrahim, Tuba Esatbeyoglu, Taha Mehany

**Affiliations:** 1 Department of Food Technology, Arid Lands Cultivation Research Institute, City of Scientific Research and Technological Applications, New Borg El-Arab 21934, Egypt; 2 Department of Food Hygiene and Control, Faculty of Veterinary Medicine, Alexandria University, Alexandria 21544, Egypt; 3 Department of Botany and Microbiology, Faculty of Science, Kafrelsheikh University, Kafr El Sheikh 33516, Egypt; 4 Department of Food Toxicology and Contaminants, National Research Centre, Dokki, Cairo 12622, Egypt; 5 Technical University of Munich Campus Straubing for Biotechnology and Sustainability, Essigberg 3, 94315 Straubing, Germany; 6 German Institute of Food Technologies (DIL e.V.), Prof.-von-Klitzing-Straße 7, 49610 Quakenbrück, Germany; 7 Department of Veterinary Forensic Medicine and Toxicology, Faculty of Veterinary Medicine, Alexandria University, Alexandria 21544, Egypt; 8 Department of Microbiology, Medicine/Alexandria University Branch, Matrouh University, Marsa Matruh 51511, Egypt; 9 Department of Microbiology, Faculty of Agriculture, Mansoura University, Mansoura 35516, Egypt; 10 Food and Nutritional Sciences, North Carolina Agricultural and Technical State University, E. Market Street 1601, Greensboro, NC 24711, USA; 11 Department of Food Development and Food Quality, Institute of Food Science and Human Nutrition, Gottfried Wilhelm Leibniz University Hannover, Am Kleinen Felde 30, 30167 Hannover, Germany

**Keywords:** aflatoxin, biocontrol, gastrointestinal tract, natural adsorbent, probiotic, *Saccharomyces cerevisiae*, *Lactobacillus rhamnosus*, chocolate contamination, chocolate safety

## Abstract

The high incidence of aflatoxins (AFs) in chocolates suggests the necessity to create a practical and cost-effective processing strategy for eliminating mycotoxins. The present study aimed to assess the adsorption abilities of activated charcoal (A. charcoal), yeast (*Saccharomyces cerevisiae*), and the probiotic *Lactobacillus rhamnosus* as AFs adsorbents in three forms—sole, di- and tri-mix—in phosphate-buffered saline (PBS) through an in vitro approach, simulated to mimic the conditions present in the gastrointestinal tract (GIT) based on pH, time and AFs concentration. In addition, the novel fortification of chocolate with A. charcoal, probiotic, and yeast (tri-mix adsorbents) was evaluated for its effects on the sensory properties. Using HPLC, 60 samples of dark, milk, bitter, couverture, powder, and wafer chocolates were examined for the presence of AFs. Results showed that all the examined samples contained AFs, with maximum concentrations of 2.32, 1.81, and 1.66 µg/kg for powder, milk, and dark chocolates, respectively. The combined treatment demonstrated the highest adsorption efficiency (96.8%) among all tested compounds. Scanning electron microscope (SEM) analysis revealed the tested adsorbents to be effective AF-binding agents. Moreover, the novel combination of tri-mix fortified chocolate had a minor cytotoxicity impact on the adsorptive abilities, with the highest binding at pH 6.8 for 4 h, in addition to inducing an insignificant effect on the sensory attributes of dark chocolate. Tri-mix is thus recommended in the manufacturing of dark chocolate in order to enhance the safety of the newly developed product.

## 1. Introduction

The history of chocolate dates back more than 4000 years, with the primary ingredient being cocoa powder produced from cocoa (*Theobroma cacao*) beans. Fungi can easily contaminate cocoa beans during the product-handling stages that include harvesting, drying, fermentation, roasting, preparation, transport, and storage. However, these steps are crucial for the formation of a distinctive chocolate flavour. Although this process is adequate for the elimination of harmful bacteria and molds, it has been demonstrated that mycotoxins are able to maintain their stability during most heat processing. Moreover, unhygienic practices can occur during the manufacturing of cocoa powder and chocolates [1]. Mycotoxin contamination from mycotoxigenic fungi such as *Penicillium*, *Aspergillus*, *Fusarium* and *Alternaria*, is thus a critical hazard to food quality and safety, with their occurrence in foodstuffs estimated to be 60–80% and resulting in over US $ 932 million in economic losses each year in the agro-food sector [2]. Aflatoxins (AFs) are the most frequently identified mycotoxins in cocoa, and the optimal conditions for AFs production by *Aspergillus* species in cocoa are at 33 °C and 0.99 a_w_ [3]. The ingestion of foods contaminated with AFs can result in severe side effects, including genetic disorders, tumors, mutagenicity, carcinogenic effects, and cytotoxicity [4]. 

According to Li et al. [5], a technical adsorbent is described as an agent that triggers the elimination of mycotoxins via adsorption or binding. Thus, the addition of adsorbents to contaminated food is a progressive and secure method for reducing the detrimental health impacts of AFs. However, the majority of adsorbents have significant practical restrictions. Activated charcoal (A. charcoal) is used as an antidote against severe poisoning. In this regard, and according to the German Federal Statistical Office (GFSO) in 2016, 178,425 cases of intoxication poisoning were treated in German hospitals. A. charcoal was recommended in 4.37% of cases. A. charcoal employment plays a major role in both primary and secondary detoxification. Moreover, in 2013, A. charcoal was used in treatment in 0.89% of cases of poisoning in children, for cases registered in the US. Therefore, A. charcoal is indicated to treat moderately severe to life-threatening intoxication [6]. A. charcoal has an excellent adsorbent property due to its porosity and large surface area. As a result, it can remove harmful pollutants such as harmful gases, heavy metals, mycotoxins, pesticides, and other chemicals from aqueous solutions [7].

The maximum ratios for mycotoxins in foods are very low owing to their severe toxicities. For instance, the maximum levels for AFs set by the Codex in various grains, seeds, nuts, milk and dried figs are in the range between 0.5–15 µg/kg (a 1 µg is 1 billionth of a kilogram) [8]. Moreover, Brazilian Sanitary Surveillance Agency has set limits of 10 μg/kg for cocoa beans and 5 μg/kg for cocoa products and chocolate sold in Brazil, for both ochratoxin A and total aflatoxins [9].

Chemical agents such as curcumin can be utilized to eliminate the mycotoxins in vivo and could antagonize the deleterious effects of AFB_1_ on the kidney in mice. This impact is realized by inhibiting Bax/Bcl-2–Cyt-c signaling cascade-mediated apoptosis and modulating the Keap1–Nrf2 signal mechanism in order to improve renal antioxidant activity. These findings confirmed the application of curcumin as a natural food additive to degrade AFB_1_ [10]. Moreover, enzymes could be a promising degrading candidate against aflatoxins toxicity. In this regard, Zhou et al. [11] developed a new laccase which purified from the white-rot fungus (*Cerrena unicolor*) in order to catalyze AFB_1_ degradation. AFB_1_ elimination by laccase was performed at 45 °C/24 h and pH 7.0 in vitro. The half-life of AFB_1_ degradation catalyzed by laccase was 5.16 h; 2,2′-azino-bis-(3-ethylbenzothiazoline-6-sulfonic acid), Syringaldehyde, and Acetosyringone, at 1 mM concentration, seemed to be similar mediators for greatly improving AFB_1_ detoxification by laccase. These results are promising for a potential application of laccase as a novel aflatoxin oxidase in degrading AFB_1_ in both feeds and foods.

Recently, there has been a growing interest in the use of probiotics and other natural alternatives that do not rely on chemical additives to counteract the toxins and pollutants in foods. For example, probiotics are useful in food bioremediation due to their excellent antioxidant potential and antimicrobial properties [12,13], in addition to being an eco-friendly, highly effective, valuable technology in food processing. Probiotics are defined as microorganisms that have a positive effect on human health when consumed in sufficient quantities. *Lactobacillus rhamnosus* and *Saccharomyces cerevisiae* are examples of generally recognized as safe (GRAS) microorganisms; consequently, they can be used as feed additives with minimal risks [14]. Moreover, for decontamination purposes, lactic acid bacteria (LAB) may be effective at decreasing mycotoxins in alcoholic drinks during processing [15]. Similarly, *S. cerevisiae* has been considered to be a food additive for decades as it plays a fundamental role in providing vitamin B, minerals, and proteins [16]. *S. cerevisiae* is currently of considerable importance with regard to several biotechnological applications. For example, the biotechnology benefit of *S. cerevisiae* is inherent in its unique biological properties, such as its fermentation ability, conveyed by the production of CO_2_ and alcohol, and its flexibility to opposing circumstances of low pH and osmolarity. The most prominent applications involving the utility of *S. cerevisiae* are in the food, beverage, and biofuel production industries [17].

LAB can thus eliminate or remove mycotoxins in food by either physical attachment or bio-transforming mechanisms [18]. The adsorption efficiency of A. charcoal and lactic acid bacteria in the removal of AFs from liquid solutions was recently determined in several studies [19,20]. In another study, it was found that certain probiotics can adsorb AFs in doogh (cultured dairy product) during fermentation and storage. The adsorption rate depends on the type of probiotics. Thus, the use of *Lactobacillus* strains such as *L. acidophilus*, *L. rhamnosus*, and *L. casei* for the ripening of cocoa has been evidenced to be an approach with excellent prospects [21].

Currently, there is no commercially available chocolate containing tri-mix (charcoal, probiotic, and yeast) and nothing in the current literature addresses such food formulation. In addition, to the best of our knowledge, no investigation has described the use of adsorbent combination models for sequestering AFs in food matrices. Consequently, the aim of this study was to evaluate and compare the effectiveness of A. charcoal, *L. rhamnosus*, and *S. cerevisiae* as aflatoxin adsorbents in three forms—sole, di- and tri-mix—in phosphate buffered saline (PBS) using an in vitro approach simulated to mimic the conditions found in the gastrointestinal tract (GIT) based on pH, time and AFs concentration. In addition, the adsorptive activity of tri-mix was studied using an in vitro dark chocolate model, and its effect on the sensory properties of novel tri-mix-fortified chocolate was evaluated.

## 2. Results

### 2.1. Screening of Aflatoxins in Various Chocolate Kinds

A total of 60 local chocolate samples of six chocolate types (10 samples each) were surveyed for the presence of total and individual AFs. The occurrence and concentrations of the tested AFs in chocolate samples are summarized in Table 1. The results demonstrated that all chocolate samples were contaminated by AFs. The highest concentration of total AFs was observed in chocolate powder when compared with other chocolate types in the following order: Chocolate powder: 2.32 > milk: 1.81 > dark: 1.66 > bitter: 0.704 > wafer: 0.674 > coverture: 0.290 µg/kg. The contamination rates of AFs in powder, milk and dark chocolates are significantly higher (*p* < 0.05) than those in other chocolates. The concentrations of AFB were higher than AFG in all examined chocolates. In order, the AFB_1_ contents in milk, dark, wafer, and bitter chocolates were 0.966, 0.963, 0.414, and 0.369 µg/kg, respectively, while the least AFB_1_ concentration presented in coverture chocolate, with 0.112 µg/kg. The contamination rates of AFB_1_ in powder, dark, and milk chocolates are significantly different (*p* < 0.05) from other chocolates. Regarding the AFB_2_ level, its concentration reached the peak in chocolate powder with a mean of 0.593 µg/kg, while its concentrations were slightly lower in the dark, bitter, milk, and wafer, at 0.573, 0.304, 0.239, and 0.207 µg/kg, respectively. The lowest concentration was reported in coverture chocolate (0.071 µg/kg). In this study, the magnitude of AFG demonstrated different levels among examined chocolates with lower values than AFB. In this context, milk chocolate showed the highest mean concentrations of AFG_1_ and AFG_2_, which were 0.425 and 0.178 µg/kg, respectively. The levels of AFG_1_ and AFG_2_ in chocolate powder were: 0.399 and 0.164 µg/kg > dark chocolate; 0.069 and 0.052 µg/kg > coverture chocolate; and 0.067 and 0.041 µg/kg, respectively. In contrast, the lowest AFG levels were reported in wafer and bitter chocolate to be 0.043 and 0.013 µg/kg for AFG_1_ and 0.010 and 0.18 µg/kg for AFG_2_, respectively.

### 2.2. Aflatoxin Adsorption Efficiency in Phosphate Buffer Solution 

The results of adsorption efficiency of (A. charcoal + *L. rhamnosus* + *S. cerevisiae*) in sole, di-mixed, and tri-mixed PBS solution against AFs (B_1_, B_2_, G_1_, and G_2_) under pH conditions (3 and 6.8) and at 2-time intervals (2 and 4 h) are illustrated in Table 2. The results were compared with the negative control (PBS), which showed the AF adsorption and residual levels of zero and positive control (PBS + AFs), with AF adsorption (zero%) and residual levels (0.99 and 1.00 μg/mL). All the tested compounds were able to adsorb AFs in PBS with varying degrees. In this study, the combined formula (tri-mix) showed higher adsorption efficiencies (*p* < 0.05) and lowered residual AFs levels than individual tested constituents. This finding reflects the synergistic actions between adsorbents against AFs in the combined tri-mix. Accordingly, A. charcoal + *L. rhamnosus* + *S. cerevisiae* tri-mix revealed the greatest AF adsorption of 96.8% and 97.7% at pH 3 and 6.8 for 2 h, respectively, while the values were 98.1% and 99.7% at pH 3 and 6.8 for 4 h, respectively. On the contrary, the concentrations of residual AFs were (0.032–0.019 μg/mL) at pH 3 and (0.023–0.003 μg/mL) at pH 6.8 after 2 and 4 h, respectively. Concerning the di-mix tested compounds, we noticed that A. charcoal + *S. cerevisiae* di-mixed PBS demonstrated the highest AF adsorption (89% and 91% for 2 and 4 h, respectively, at all pH conditions) as well as the lowest residual AFs levels (0.10 and 0.09 μg/mL for 2 and 4 h, respectively, at all pH conditions), compared with *L. rhamnosus* + *S. cerevisiae* and A. charcoal + *L. rhamnosus* di-mixed PBS. The AF adsorption values for *L. rhamnosus* + *S. cerevisiae* vis A. charcoal + *L. rhamnosus* di-mixed buffer were 83% and 85% vis 80% and 78% at pH 3, while at pH 6.8, the values were 85% and 84% versus 80% after 2 and 4 h, respectively. In contrast, the residual AFs levels were 0.17 and 0.15 versus 0.20 and 0.22 μg/mL at pH 3, as well as 0.15 and 0.16 versus 0.20 μg/mL at pH 6.8 after 2 and 4 h, respectively, for *L. rhamnosus* + *S. cerevisiae* versus A. charcoal + *L. rhamnosus* di-mixed PBS.

### 2.3. Aflatoxin Adsorption Efficiency in Fortified Dark Chocolate

An experiment on the adsorption of AFs by the tri-mix (A. charcoal + *L. rhamnosus* + *S. cerevisiae*) was carried out in model chocolate to assess the AF adsorption efficiency of the tri-mix. The experiment was conducted at two time intervals (2 and 4 h) under pH conditions (3.0 and 6.8). As depicted in Table 3, the overall results indicate a significant reduction in AFs in supplemented chocolate compared to negative (chocolate) and positive control (chocolate + AFs). In the positive control, there was a consistency in the range of total AFs (0.98–0.99 µg/kg), with zero adsorption throughout the time intervals and pH conditions. In contrast, the adsorption efficiency at simulated gastric pH (3.0) was better, with an adsorption of 95.4% and 96.1% at 2 and 4 h, respectively, while the levels of residual total AFs were 0.046 and 0.039 µg/kg at 2 and 4 h, respectively. The highest adsorption and the lowest residual total AFs were achieved at pH 6.8 after 4 h, at 96.80% and 0.032 µg/kg, respectively. In contrast, the lowest adsorption and the highest residual total AFs were 90.2% and 0.098 µg/kg, respectively, after 2 h at the same pH. Regarding the adsorption effect on the type of AFs, we found that AFG_1_ was the most adsorbed AF in supplemented chocolate when compared with other AFs, with residual AFG_1_ values of 0.004 and 0.002 µg/kg at pH 3.0 and 0.003, respectively, and 0.001 µg/kg at pH 6.8, when compared with the range of positive control (0.24–0.25 µg/kg). Furthermore, all AFs types were strongly adsorbable at pH 6.8 compared with pH 3.0, for 2 and 4 h.

### 2.4. Scanning Electron Microscope Assessment

**Figure 1:**
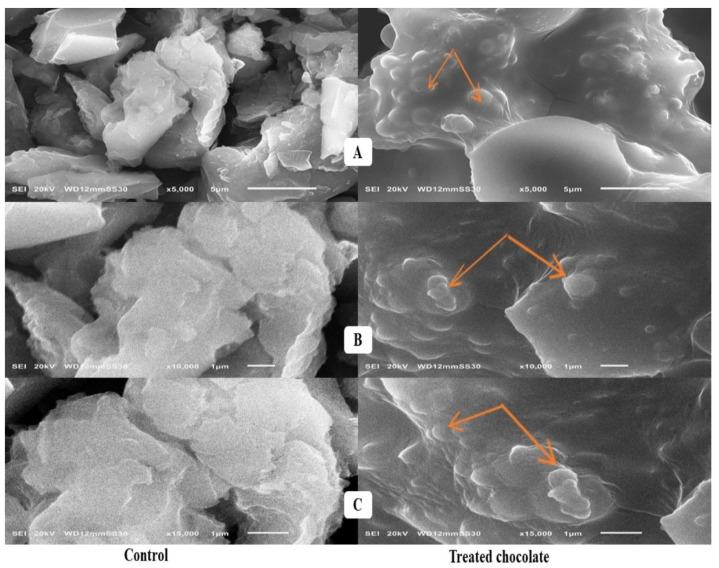
Scanning electron microscopy analysis of activated charcoal + AFs mix. (**A**–**C**) with 5000, 10,000, and 15,000× magnifications, respectively: control group (untreated charcoal in the left side with honeycomb vacuumed structure) and treatment group (charcoal incubated with 1 mg/mL of AFs for 4 h in the right side). Orange arrows denote the AF surplus upper layer and pimples on the surface of activated charcoal.

The microscopic examination has been applied to determine the surface characteristics of A. charcoal, probiotics, and yeast cells before and after AF adsorption. Drastic changes in the surface morphology of A. charcoal after incubation with AFs mix (B_1_, B_2_, G_1_, and G_2_) for 4 h were observed (Figure 1) when compared with non-incubated A. charcoal (negative control).

**Figure 2:**
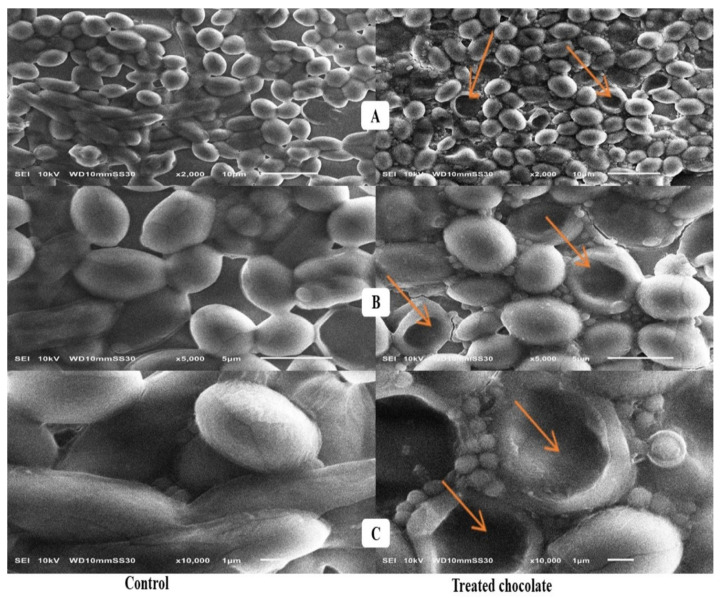
Scanning electron microscopy analysis of *S. cerevisiae* cells + AFs mix. (**A**–**C**) with 2000, 5000, and 10,000× magnifications, respectively: control group (untreated cells in the left side having diploid form and ellipsoid-shaped) and treatment group (*S. cerevisiae* cells incubated with 1 mg/mL of AFs for 4 h in the right side). Orange arrows indicate structural changes induced by Afs, with an additional upper layer of AFs and their perforations. The yeast cells appeared spheroid with deformations and cavitation in the cell wall.

In the SEM investigation (Figure 2), with 2000, 5000, and 10,000× magnifications, the untreated *S. cerevisiae* cells, having a diploid form and ellipsoid shape, are observed with capsules of a typical size of 1.71 ± 0.03 mm. The treated cells appeared spheroid, with deformations and cavitation in the yeast cell wall.

**Figure 3:**
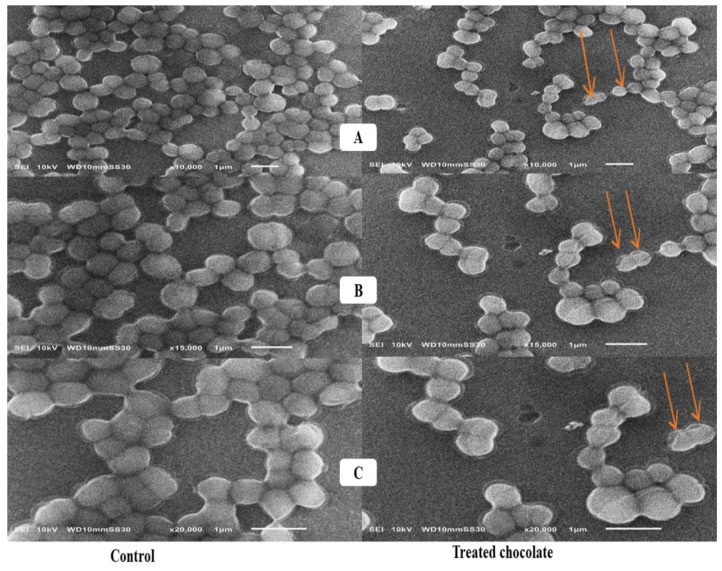
Scanning electron microscopy analysis of *L. rhamnosus* cells + AFs mix. (**A**–**C**) with 10,000, 15,000, and 20,000× magnifications, respectively: control group (untreated rod-shaped cells in the left side arranged in chains) and treatment group (*L. rhamnosus* cells incubated with 1 mg/mL of AFs for 4 h in the right side). Orange arrows refer to morphological alterations made by AFs; the bacterial cells appeared as bud-like structures.

In the typical micrograph of SEM (Figure 3), with 10,000, 15,000, and 20,000× magnifications, the untreated *L. rhamnosus* cells (control) appeared as small undamaged rods arranged in chains. The SEM imaging reveals a prevalent conformational change probably produced by AFs attached to the cell wall surface.

### 2.5. Cytotoxicity Assessment of Activated Charcoal 

Table 4 illustrates the cytotoxicity assessment of charcoal to determine the IC_50_ (µg/mL) on peripheral blood mononuclear cells (PBMCs). Carbon particles have been proven to be toxic to PBMCs at various concentrations. Consequently, cells exposed to A. charcoal were presented with 94–47% of live cells at concentrations less than 48.8 µg/mL, while at a higher concentration, above 97.5 µg/mL, the cells demonstrated 18–29% of live cells.

### 2.6. Sensorial Properties of Tri-Mix-Fortified Dark Chocolate 

In the sensory evaluation of dark chocolate, taste and texture were the most important characteristics, particularly in the tri-mix-fortified chocolate compared with the control. The results in Table 5 reveal that all sensory scores are not significantly different between chocolates, except for taste and texture. The mean values of taste and texture for tri-mix-chocolate were equalized to be 8.0, which was lower than those for the control (8.6). Commercially, A. charcoal has porous and gritty nature, which may affect the texture of chocolate. The mean values of odor and overall acceptance for tri-mix chocolate versus control were 8.1 versus 8.5, and 8.3 versus 8.6, respectively.

## 3. Discussion

### 3.1. Screening of Aflatoxins in Chocolates

AFB_1_ was the most frequently detected aflatoxin at the highest concentration, compared with AFB_2_, AFG_1_, and AFG_2_, in all chocolates. Accordingly, the highest mean concentration of AFB_1_ was detected in chocolate powder, at 1.12 µg/kg, exceeding the permissible limit according to Codex Standard (1 µg/kg) [22]. On the other hand, our findings regarding AFs in all tested chocolate are within the allowable limits of the Brazilian Sanitary Surveillance Agency, which set limits of 10 μg/kg for cocoa beans and 5 μg/kg for cocoa products and chocolate sold in Brazil, for both ochratoxin A and total aflatoxins [8].

The contamination levels of AFG in powder and milk chocolates are significantly different (*p* < 0.05) from other chocolates. This finding is inconsistent with those reported by Kabak [23], who found that AFG_1_ and G_2_ contents failed to be detected in all chocolate samples, except milk chocolate, where the concentration of AFG_1_ was 0.35 μg/kg.

These results are relatively similar to those obtained by Copetti et al. [24] who detected AFs in approximately 72% of milk chocolates and 100% of bitter and dark chocolate at concentration ranges of 0.11–1.65 and 0.04–0.91 μg/kg, respectively. In a study by Turcotte et al. [25] the AFB_1_ was reported in 80% of dark chocolate and 70% of milk chocolate samples at values of 0.63 and 0.18 μg/kg, respectively. In Pakistan, Naz et al. [26] reported that the contents of AFs in dark, milk, and bitter chocolates were 2.27, 1.31, and 0.97 µg/kg, respectively. In contrast, our findings are significantly higher than those reported by Kabak, [23] who counted AFs in 19.6% of milk chocolate, 13.3% of bitter chocolate, and 8.70% of chocolate wafer samples at mean values of 0.839, 0.455, and 0.281 μg/kg, respectively. The authors confirmed that AFB_1_ was the highest detected AF in all examined chocolate samples.

To date, there is no maximum threshold for AFs in cocoa and its products involving chocolate established by the European Regulation. The differences in AFs levels amongst examined chocolates may indicate the content and quality of cocoa solids. This finding may explain the high content of total Afs, 2.322 and 1.658 µg/kg, in cocoa powder and dark chocolate samples containing 100% and 35% cocoa solids, respectively. Although milk chocolate sample content was 29–35% cocoa solids [22], the high content of total AFs (1.81 µg/kg) could be attributed to milk solids, based on the presence of AFs in milk products reported recently [27]. The higher contamination levels of chocolates containing AFs may be attributable to the deficiency of quality control and quality assurance during the manufacturing, and unhygienic packaging. Currently, chocolate consumption among Egyptians, particularly children, is high. Consequently, quantifying the presence of mycotoxins in these products is a significant issue, and additional research is required to determine their mycotoxin concentrations.

### 3.2. Aflatoxin Adsorption Efficiency in Phosphate Buffer Solution

By comparing the individual tested compounds, it was found that *S. cerevisiae* adsorbed the highest levels of AFs with adsorption vis residual levels of 71% vis 0.29 μg/mL and 73% vis 0.27 μg/mL at pH 3.0, and 71% vis 0.29 μg/mL at pH 6.8, after 2 and 4 h, respectively. This result is higher than those explained by Istiqomah et al. [28], who found that the AFs binding activity of autoclaved *S. cerevisiae* B18 cells was 69.5%. Additionally, Rahaie et al. [29] illustrated that the heat treatment of yeast cells increased their binding abilities to AFs by 56 ± 2%. A. charcoal adsorbed 52–55% (pH 3.0) and 52% (pH 6.8) of AFs versus 52–53% (pH 3.0) and 51% (pH 6.8) for *L. rhamnosus*, after 2 and 4 h, respectively. This finding indicates that *L. rhamnosus*-supplemented PBS demonstrated the highest levels of residual Afs, which were 0.48 and 0.47 μg/mL at pH 3.0, and 0.49 μg/mL at pH 6.8, for 2 and 4 h, respectively.

This investigation suggests that the adsorption efficiency depends on several reasons, e.g., physical, chemical, and biological properties of the adsorbent and the adsorbate (the food or beverage); concentration of the adsorbate in fluids; features of the liquid phase (e.g., pH, temperature) and the residence time. The tri-mix demonstrated the maximum AF adsorption, followed by the di-mix, due to the synergism among the tested adsorbents’ physical, chemical, ion-exchanging, and biological mechanisms. Accordingly, A. charcoal has a large surface area and pore volume, providing an excellent adsorption capability. It can sequester molecules, including mycotoxins, via both chemical and physical interactions; hence, it can be used as an adsorbent agent. A. charcoal was recognized to have binding efficiency of more than 90% with AFs.

*Lactobacillus* strains are Gram-positive bacteria, with a peptidoglycan layer in their cell wall and teichoic acid and polysaccharides (β-D-glucan) on the surface. The divalent cations can lead to alterations in the teichoic acid structure. Furthermore, the adsorption ability of lactobacilli is generally strain-dependent, due to the differences in the structure of biochemical components on the cell wall for most probiotic types [30,31].

In this study, we found that all the PBS-tested compounds containing *S. cerevisiae* demonstrated the best AF adsorption. The adsorption capability mainly depended on yeast composition and mycotoxin. Earlier studies investigated that yeast cells had the ability to attach numerous molecules involving mycotoxins via the polysaccharides (glucans), mannoproteins, and lipids found on their cell wall surface [32]. They can bind to the hydroxyl, ketone, and lactone groups of AFs by hydrogen bonds and van der Waals forces [33]. Furthermore, the natural characteristics of mycotoxins play an integral role in the adsorption activities, such as polarization, solubility, size, shape, cation exchange capacity (CEC), acidity, and relative humidity [34]. This finding agrees with Joannis-Cassan et al. [35], who indicated that the yeast cell wall components played a crucial role in AFs binding by *S. cerevisiae*. Furthermore, *S. cerevisiae* was the most effective microorganism for binding AFs in PBS. Concerning the synergetic action achieved in this study, A. charcoal displays reversible physical adsorption for microorganisms in liquids without removal by simple desorption and permeability, permitting its usage as a bio preservative [3]. A study by Ikegamai et al. [36] revealed that the fermentation activities of *S. cerevisiae* reached 90% in the medium containing A. charcoal, compared to 70% in controls. Besides, attractive interactions between microorganisms and A. charcoal decreased the porosity and negative charges, enhancing the capacity of A. charcoal to adsorb AFs, which might be the chief cause of maximum adsorptive ability for the tri-mixed PBS solution.

As hypothesized, the AF adsorption varied with the type and initial concentration of aflatoxin. Accordingly, AFB_1_ was the greatest adsorbed aflatoxin on A. charcoal + *L. rhamnosus* + *S. cerevisiae*-treated PBS, with residual values of 0.003 ± 0.01 μg/mL at pH 6.8 for 4 h, compared with the initial concentration of 0.24 ± 0.01 μg/mL. Furthermore, AFB_2_ achieved the best adsorption on A. charcoal + *L. rhamnosus*+ *S. cerevisiae*-treated buffer at pH 3 for 4 h compared to other Afs, with a residual level of 0 μg/mL of an initial concentration of 0.25 ± 0.01 μg/mL. Similar findings were obtained by El-Nezami et al. [37] who found that *L. rhamnosus* strains successfully bound AFB_1_ and B_2_, rather than G_1_ and G_2_. In contrast, Liew et al. [20] showed that the cell wall of lactobacilli showed the highest adsorption, at 97% for an AFB_1_ concentration of 6 µg/mL. Similarly, Hernandez-Mendoza et al. [38] investigated the feasibility that teichoic acid contributes to AFB-binding by lactobacilli. The ability of bacterial cells to bind with AFs could be enhanced by chemical conditions, as bacterial cell treatment with acid facilitated the AFs’ physical binding to the bacterium’s molecular components, especially at the cell wall level.

With respect to AFG_2_, it was the most efficiently absorbed aflatoxin by *S. cerevisiae*, and A. charcoal + *S. cerevisiae* treated PBS compared to other AFs with residual values of 0.07 ± 0.01 and 0.02 μg/mL, respectively. Moreover, AFG_1_ and G_2_ showed a relatively lower residual level (0.005 ± 0.002 μg/mL) on A. charcoal + *L. rhamnosus* + *S. cerevisiae* tri-mixed PBS when compared with AFB_1_ and B_2_ on the same buffer. All AFs showed the same residual values on A. charcoal + *L. rhamnosus*-treated PBS to be 0.04 ± 0.01 μg/mL.

The adsorption efficiency in this study is higher than those recorded by Shetty et al. [39], who examined the adsorption of AFB_1_ by 18 species of Saccharomyces at AFB_1_ concentrations of 1, 5, and 20 µg/mL. The authors reported that the yeast cells adsorbed 69.1% of the AFB_1_ at 1 µg/mL, 41.0% at 5 µg/mL of AFB_1_, and 34.0% at 20 µg/mL of AFB_1_. In this study, an increase in adsorption was achieved at a reduced initial concentration of each AF, around 0.25 ± 0.01 μg/mL, so the adsorption levels were relatively high. These findings are compatible with Joannis-Cassan et al. [35] who reported that the range of adsorption was 2.5% to 49.3%, based on the AFB_1_ concentration and the adsorbent type. Similarly, Gallo and Masoero [40] reported that the adsorption varied from 32% to 54%, with an initial AFB_1_ concentration of 0.82 µg/mL.

By modifying surface charges and adsorbent–adsorbate reactions, pH substantially affects a liquid medium’s adsorption efficiency. Hence, the adsorption ability of all adsorbents for the AFs was evaluated at 2 pH levels of 3.0 and 6.8. In this regard, we found that the only treatment of PBS either by A. charcoal or L. rhamnosus, or S. cerevisiae achieved the greatest adsorption, ranging from 52% to 73% at acidic pH (3.0). In a study by Joannis-Cassan et al. [35] they demonstrated that acidic pH (3.0) permitted adequate adsorption of the mycotoxins in the buffer. Exchangeable cations are involved in the binding mechanisms of AFs, which can be enhanced after acidulating the adsorbent surface.

In contrast, the di-mixed treatment of PBS by A. charcoal + *L. rhamnosus*, A. charcoal + *S. cerevisiae*, or *L. rhamnosus* + *S. cerevisiae* demonstrated the best adsorption, varying from 80% to 91% at pH 6.8. The tri-mixed PBS by A. charcoal + *L. rhamnosus* + *S. cerevisiae* had the overall maximum adsorption of 97.7% and 99.70% at pH 6.8. These results indicate that the adsorption of AFB_1_ and AFB_2_ by the combined adsorbents was pH-independent, whereas adsorption of AFG_1_ and AFG_2_ increased to a certain extent at neutral pH (6.8), as evidenced by the net surface charges on adsorbents and the charge on AFs molecules. Similar findings were reported by Rasheed et al. [41] who found no evident variation in the adsorption for AFB_1_ and AFB_2_ in buffer at pH 7, while for AFG_1_ and AFG_2_, adsorption increased enormously. Moreover, this result agrees with Tejada et al. [42] who noticed that the highest adsorption of A. charcoal reached a value of 75.4% in a solution at pH 6.

In the present research, we used two time intervals, and the adsorption efficiency of AFs by all treated buffers was more significant at 4 h than at 2 h. This result is compatible with Rahaie et al. [29], who confirmed that the adsorption capability of AFs in a buffer may occur at 2 h to 3 h of treatment. The variation in the incubation time could be the factor that influences the adsorption efficiency of AFs. In the current study, the incubation times of ranging from 2–4 h were used to reflect the transit time in human GIT. In addition, the absorption of AFs mainly occurs in the small intestine [19]. Generally, incubation time plays a part in the adsorption activity.

### 3.3. Aflatoxin Adsorption Efficiency in Supplemented Dark Chocolate

Although the results of AF adsorption by the tri-mix in the supplemented chocolate were high, they were relatively lower than in the PBS solution. This decreased percentage suggests chocolate has a lower cation exchange capacity (CEC) than the PBS medium. Additionally, this concept could be acceptable due to polyphenols and alkaloids in the model chocolate having great affinity for less polar AFs and acting as an ideal solvent for them [43], therefore affecting the adsorption efficiency between AFs and adsorbents. Additionally, the hydrophilic proteins involved in the composition of chocolate might compete with AF molecules for adsorption sites on adsorbents, thus decreasing its adsorption capacity. This finding agrees with Barrientos-Velázquez et al. [43]; they found that proteins blocked the hydrophobic sites needed for Afs on the surface of the adsorbent. The results of AF adsorption in model chocolate revealed marginal dissimilarity, which could be due to the presence of a distribution coefficient for AFs amongst aqueous and solid phases compared with PBS.

### 3.4. Scanning Electron Microscope Assessment

The non-incubated sample exhibited a honeycomb vacuumed morphology similar to the turbostratic shape of the carbon molecules. This finding reflects the large surface area of the carbon particles. The same result was obtained by Kalagatur et al. [44]. The incubated sample showed a highly exfoliated and rougher surface after AF adsorption. The honeycomb shape was replaced by a pimple-like structure, due to an additional upper layer of AFs appearing on the particle surface of A. charcoal.

Vilela et al. [45] informed that such holes can be formed by the action of adsorption, as yeasts are normally characterized by their egg shape and the smoothness of the surface. With a magnification of 15,000×, it is noticed that the capsule’s core has a slight irregularity with holes along its whole length, which means that the internal morphological structure and the surface of holes were available to the molecules of AFs. Similar results of specific surface area were reported by De Rossi et al. [46].

The appearance of bud-like structures on the surface of bacterial cells was only noticed in the AFs-treated cells. Besides, the morphological changes involved the asymmetrical and coarse surface of the bacterial cell wall. It can be assumed that the interaction between AFs and the teichoic acids surface and beta-d-glucan structure of the *L. rhamnosus* cell wall might cause the structural variations noticed in SEM imaging. The same result of morphological alterations on the bacterial surface against AFB_1_ was observed by Liew et al. [20].

### 3.5. Cytotoxicity Assessment of Activated Charcoal 

The results of cytotoxicity in the current research contradicts the findings of Kalagatur et al. [44], who reported the non-toxic effect of A. carbon against neuro-2a cells, displaying 95.46% of live cells. The toxic effect may result from the size and shape, porosity, surface functionality, surface conductivity, or, frequently, the toxic guest species allied with these materials during processing [47]. The IC_50_ value of A. charcoal was 45.9 µg/mL, and this result was considered in the application process, as added A. charcoal did not exceed 40 μg/g in dark chocolate.

### 3.6. Sensorial Properties of Tri-Mix-Fortified Dark Chocolate 

In the present study, dark chocolate exhibited grades of (much-like > 8.0) for all sensory attributes before and after adding tri-mix. This finding disagrees with Ronald and Normalina [48], who found that the supplementation of dark chocolate cake with A. charcoal enhanced its odor and overall interpretation. As hypothesized, A. charcoal acts as a taste- and odor-eliminating agent, so it might adsorb some of the volatile compounds responsible for the aroma and taste perception of dark chocolate. The color and appearance ratings showed the same values of 8.4 and 7.9 in both chocolates, which might be due to the black color of the A. charcoal hidden with the color of the dark chocolate, enhancing its color. This result agrees with Ronald and Normalina [48] who recorded a much-accepted appearance of dark chocolate cake after adding charcoal. This study determined that the tri-mix-fortified chocolate had a negligible impact on the adsorptive abilities of the tri-mix. Therefore, we suggest the application of tri-mix in manufacturing dark chocolate. The amount of A. charcoal used in this evaluation was ideal as it caused a minimal effect on the sensory scoring of dark chocolate. We should consider that A. charcoal is a relatively unspecific adsorbent; hence, essential dark chocolate nutrients could also be adsorbed particularly if its concentrations in the food are much greater than those of the AFs.

## 4. Conclusions

In the present study, it was shown that powder, dark, and milk chocolates contained the highest concentrations of aflatoxins, recommending the implementation of good agricultural practices (GAPs) in cocoa farms, and good manufacturing practices (GMPs) in all steps of chocolate processing. In vitro assessment of adsorption abilities of A. charcoal, *S. cerevisiae*, and *L. rhamnosus* revealed that combined adsorbents exhibited higher adsorption efficacy toward AFs than that of individual adsorbents. The highest binding (96.80%) was reported for tri-mix (A. charcoal+ *S. cerevisiae* +*L. rhamnosus*) at neutral pH 6.8 for 4 h. The SEM micrographs showed that AFs caused structural changes on the surfaces of all adsorbents with extraordinary trapping. The dark chocolate newly fortified with tri-mix (A. charcoal+ *S. cerevisiae* +*L. rhamnosus*) displayed a maximum removal of AFs and a high adsorptive power with minimal effects on its sensory evaluation. As a result, tri-mix has significant potential to be extensively utilized as a bio-functional food for enhancing the safety of this newly developed chocolate model. Moreover, further investigations to address various chocolate types produced from different countries and based on spectroscopic techniques combined with big data analyses are highly recommended.

## 5. Materials and Methods

### 5.1. Chemicals and Reagents 

All reagents and cultural media used in the current study were provided by Fluka chemicals (Fisher Scientific, Cairo, Egypt), and Merck Co (Kenilworth, NJ, USA). AFs and A. charcoal used were bought from SD Fine-Chem company, Maharashtra, India (Product No: 43032, Methylene blue adsorption: 270 mg/g, particle size: 300 mesh, pH: 6–7.5, maximum limits of impurities: ash 2.5%, moisture 5%, water-soluble 1.5%, and acid-soluble 2.5%).

### 5.2. Standards Preparation

The standard solution of AFs was prepared in methanol at concentrations of 1.00, 0.30, 1.00, and 0.30 μg/mL for AFB_1_, AFB_2_, AFG_1_, and AFG_2_, respectively. The intermediate multi-standard mixture was dissolved in methanol at concentrations of 0.10 μg/mL for AFB_1_ and AFG_1_, and 0.03 μg/mL for AFG_2_, and AFB_2_ was prepared in methanol and kept at −18 °C. Then, these solutions were utilized in the preparation of standards. The detection limits for AFB_1_, AFB_2_, AFG_1_, and AFG_2_ were 2.70, 2.50, 1.33, and 1.40 ng/mL, respectively, while the quantification limits were 3.12, 2.92, 1.61, and 1.81 ng/mL, respectively.

### 5.3. Bacterial Strains and Cultural Conditions

*Saccharomyces cerevisiae* EMCC 97 and *Lactobacillus rhamnosus* EMCC 1105 were obtained from Microbiological Resources Centre (MIRCEN), Ain Shams University, Cairo, Egypt. *S. cerevisiae* was cultured in yeast peptone dextrose medium broth (YPD) (purchased from Merck KGaA, Darmstadt, Germany), while *L. rhamnosus* was cultivated in de Man Rogosa and Sharpe broth (MRS) (obtained from HIMEDIA, Maharashtra, India) and kept overnight at 37 °C and 100 rpm. The cells were collected by centrifugation at 6000 rpm/15 min, washed with PBS, and distilled water as described by El-Nezami et al. [49]. Cells were finally suspended in 0.1 M PBS at pH (6.8), and the concentration of cells was adjusted at 600 nm to be (2 × 10^8^ CFU/mL) for *S. cerevisiae* and (1 × 10^9^ CFU/mL) for *L. rhamnosus*.

### 5.4. Screening of Aflatoxins in Various Chocolate Types 

Sixty samples of six chocolate types were purchased from various areas in Alexandria, Egypt. Ten samples of each chocolate type, dark, milk, bitter, couverture, powder, and wafer chocolates, were screened. The samples were grounded and saved at −20 °C until extraction and examination.

### 5.5. Extraction and Immunoaffinity Chromatography (IAC) Clean-Up

The extraction of AFs from chocolates was done as previously reported by Copetti et al. [24] with minor alterations. Twenty grams of chocolate were extracted by 120 mL methanol: water (8:2, *v*/*v*) and 2 g NaCl and were blended at a speed of 1500 rpm for 2 min, and then were filtered using filter paper. Six milliliters of the filtrate were mixed with 44 mL of PBS and were re-filtered through a 1.5 µm microfibre filter (VWR International, Leuven, Belgium). Sixty milliliters of the filtrate were escaped through AflaTest^®^ IAC fixed on a vacuum manifold (Agilent Technologies, Santa Clara, CA, USA) at a flow of 2 drops/second. At the same time, the column was immersed with 15 mL of Milli Q water (Siemens Ultra Clear, Germany). AFs were separated from the IAC using 0.5 mL of methanol in two stages. The collective eluates were mixed with 1 mL of Milli Q water and kept at 3–7 °C before HPLC investigation.

### 5.6. HPLC Analysis

Aflatoxin analysis of chocolate samples was applied using the Waters HPLC system (Model 6000, Milford, MA, USA), as previously reported by Hamad et al. [50]. It consists of a solvent conveyance system controller (model 720) fitted with a fluorescence detector (FLD, model 274) at 360 Ex, and 450 Em. The separation was done using a Waters regularity column (150 × 4.6 mm^2^ i.d., 5 µm), the reverse phase was utilized at a flow rate of 1 mL/min with an isocratic model consisting of 1% acetic acid: methanol: acetonitrile (55:35:10, *v*/*v*/*v*). The calibration curve was made using varied concentrations of AFB_1_, AFB_2_, AFG_1_, and AFG_2_.

### 5.7. Preparation of Adsorbents in Sole, Di, and Tri-Mix Forms

Using A. charcoal, probiotic yeast (*S. cerevisiae*), and lactic acid bacteria (*L. rhamnosus*), AF adsorption tests were conducted. The *S. cerevisiae* cell wall was prepared according to Nathanail et al. [18]. The yeast culture was activated in potato dextrose for mass culture, and then optical density was adjusted at 600 nm (T80 UV/VIS spectrophotometer, Leicestershire, UK) to reach a concentration of 2 × 10^8^ CFU/mL. Yeast pellets were collected by centrifugation at 3000 rpm/20 min (Micro centrifuge, SELECTA, Madrid, Spain), washed three times using sterilized water, then suspended in 0.1 M PBS and autoclaved at 120 °C/20 min. Finally, centrifugation was carried out at 5000 rpm/20 min, then the supernatant was wasted, and cells were washed with sterilized distilled water and freeze-dried.

The culture of *L. rhamnosus* was activated by inoculating 1 mL into 100 mL of MRS broth (Oxoid, Hampshire, UK) and was kept at 37 °C for 20 h. Then, 1 mL of this culture was then transferred to 99 mL of MRS broth to obtain a 1% dilution and incubated again was performed at 37 °C/20 h. The concentration was adjusted using a spectrophotometer at 600 nm to be 1 × 10^9^ CFU/mL. The bacterial cells were deactivated, and their cell walls were separated by incubating them with 4 mL of 2M HCl at 37 °C/1 h. The cells were then washed with 2 mL of PBS and centrifuged at 5000 rpm/10 min at 10 °C. The supernatant was then discarded, and bacterial cells were freeze-dried at −40 °C. In PBS, concentrations of 40 µg/mL of A. charcoal, lyophilized 2 × 10^8^ CFU/mL of *S. cerevisiae*, and 1 × 10^9^ CFU/mL of *L. rhamnosus* were applied individually, in pairs (di-mix) (A. charcoal + *L. rhamnosus*, A. charcoal + *S. cerevisiae*, and *L. rhamnosus* + *S. cerevisiae*), and all together (tri-mix) in PBS. The doses were prepared as follows: (1) A. charcoal = 40 µg/mL or g (2) *L. rhamnosus* = 110 µg/mL or g and (3) *S. cerevisiae* = 130 µg/mL or g.

### 5.8. Assessment of Aflatoxin Adsorption in PBS Solution

The adsorption effects of previously prepared adsorbents in the sole, di-, and tri-mix forms were examined in PBS containing aflatoxin in 2 mL tubes. The assessment was conducted at two medium pH (3.0 and 6.8) and at 2 time intervals. The samples were sited in a shaker incubator with a speed of 40 cycles/min at 37 °C/30 min. For HPLC analysis, 1 mL of the supernatant was obtained after centrifugation at 25 °C and 3000 rpm/5 min. The results were compared with those of the positive control (1 μg of AF/mL in the absence of adsorbents) and negative control (adsorbents suspended in pure PBS). Adsorption percentage was calculated according to Ghofrani Tabari et al. [51] by matching the primary aflatoxin concentrations with their concentrations in the existence of adsorbents using the following formula (Equation (1)).

(1) $$\mathrm{Adsorption}\;\mathrm{percentage}=\frac{1-\mathrm{AF}\;\mathrm{concentrations}\;\mathrm{in}\;\mathrm{the}\;\mathrm{existence}\;\mathrm{of}\;\mathrm{adsorbent}}{\mathrm{AF}\;\mathrm{concentrations}\;\mathrm{in}\;\mathrm{the}\;\mathrm{standard}\;\mathrm{sample}}\times 100$$

where AF is the aflatoxin.

### 5.9. Determination of Aflatoxins Residues Using HPLC 

A standard solution (26.0 μg/mL) of individual aflatoxin (AFB_1_, AFB_2_, AFG_1_, and AFG_2_) was prepared by liquefying 1 mL mixture of HPLC-grade benzene: acetonitrile (97:3 *v*/*v*). The standard solution was diluted until reaching a concentration of 1 µg/mL (250 ng/mL for each aflatoxin) using 0.5 mol/L PBS (pH 3.0 and 6.8). Analysis of AFs in PBS was performed according to Hamad et al. [52]. For sample derivatization, 100 µL of trifluoroacetic acid (TFA) + 200 µL of *n*-hexane were added to each sample and were vortexed for 30 s, and samples were kept for 15 min at ambient temperature. Then, 900 µL water: acetonitrile (9:1, *v*/*v*) was added and blended by a vortex. The *n*-hexane layer was discarded, and AFs in samples were determined.

### 5.10. Assessment of Aflatoxin Adsorption by Tri-Mixed Adsorbents in Dark Chocolate Model

Dark chocolate was prepared as follows: dark coverture chocolate was melted in a water bath, then after tempering at 40 °C, divided into three equal portions; (1) negative control (plain chocolate), (2) positive control (chocolate + AFs 1 µg/kg), (3) tri-mix of A. charcoal, lyophilized *L. rhamnosus* (1 × 10^9^ CFU/g) and *S. cerevisiae* (2 × 10^8^ CFU/g) with a ratio of (40:110:130 µg/kg of chocolate) and AFs mix (AFB1, AFB2, AFG1, AFG2) (1 µg/kg of chocolate). Chocolate masses were blended (1500 rpm/5 min), molded, cooled, detached from the form, wrapped in aluminum foil and paper blanks, then kept at 20 °C [53]. The adsorption effect was assessed and compared with control, after 2 and 4 h of incubation at 40 °C, at pH 3.0 and 6.8. The adsorption% and residual AFs were estimated as previously done in PBS.

### 5.11. Scanning Electron Microscope Analysis

Activated charcoal, probiotics, and yeast cell surface characteristics before and after AF adsorption were visualized by Scanning Electron Microscope (SEM) (SEM—Joel JSM 6360, LA, Tokyo, Japan).

### 5.12. Cytotoxicity Assessment of Activated Charcoal

Cell viability was examined using peripheral blood mononuclear cells (PBMCs) maintained in the RPMI medium. Blank wells (150 µL PBS), control wells (150 µL PBMCs), and tested wells (150 µL PBMCs) were allocated on a 96-well microtiter plate. Activated charcoal at different concentrations was added to test wells and incubated for 24 h. After adding neutral red (150 µL/wells), they were incubated at 37 °C/2 h, cells were washed, and plates were shaken with de-staining solution (150 µL/well) of (1% acetic acid: 49% deionized water: 50% ethanol). Absorbance was monitored at 540 nm in a spectrophotometer [54]. The percentage of inhibition = 100 − (O.D Control − O.D Treatment / O.D Control) (Equation (2)); IC_50_ values were calculated online “www.aatbio.com/tools/ic50-calculator (accessed on 14 June 2022)”.

(2) $$\mathrm{Inhibition}\;\mathrm{percentage}=100-\frac{O.D\;\mathrm{Control}\;-\;O.\;D\;\mathrm{Treatment}}{O.\;D\;\mathrm{Control}}$$

where O.D = optical density.

### 5.13. Application of Tri-Mixed Adsorbents in Dark Chocolate Model 

Dark chocolate was prepared according to Mirković et al. [53] as follows: (1) plain chocolate: control; (2) treatment: tri-mix of A. charcoal, lyophilized *L. rhamnosus* (1 × 10^9^ CFU/g), and *S. cerevisiae* (2 × 10^8^ CFU/g) with a ratio of (40:110:130 µg/kg of chocolate).

### 5.14. Sensory Evaluation

Sensory assessment of control and supplemented chocolate was carried out following the procedure of Senaka Ranadheera et al. [55] with slight modifications. The samples were examined at room temperature using a 9-point Hedonic scale for color, odor, taste, texture, appearance, and overall acceptability. Organoleptic assessment was conducted by 20 panelists (22–58 years old).

### 5.15. Statistical Analysis

Data were expressed as means of duplicates ± standard deviation (SD). Data were analyzed by multiple comparisons of one-way analysis of variance (ANOVA) using the Duncan test in IBM SPSS statistics 23 software program (IBM Corp, Armonk, NY, USA), where probability (*p* < 0.05) was considered statistically significant.

## Figures and Tables

**Table 1:** Occurrence and concentration (µg/kg) of aflatoxin residues in chocolate products.

Chocolate Products	Number of Samples	AFB_1_		AFB_2_		AFG_1_		AFG_2_		Total AFs	
		Min–Max	Mean ± SD	Min–Max	Mean ± SD	Min–Max	Mean ± SD	Min–Max	Mean ± SD	Min–Max	Mean ± SD
Couverture chocolate	10	0.015–0.542	0.112 ± 0.19 ^c^	0.006–0.262	0.071 ± 0.095 ^c^	0.014–0.096	0.067 ± 0.007 ^b^	0.013–0.086	0.041 ± 0.027 ^b,c^	0.106–0.776	0.290 ± 0.231 ^d^
Dark chocolate	10	0.06–1.87	0.963 ± 0.689 ^a^	0.076–0.972	0.573 ± 0.313 ^a^	0.036–0.098	0.069 ± 0.020 ^b^	0.037–0.062	0.052 ± 0.007 ^b^	0.247–2.669	1.658 ± 0.735 ^b^
Milk chocolate	10	0.270–1.60	0.966 ± 0.376 ^a^	0.130–0.390	0.239 ± 0.063 ^b^	0.280–0.650	0.425 ± 0.112 ^a^	0.130–0.280	0.178 ± 0.045 ^a^	1.230–2.450	1.808 ± 0.333 ^b^
Chocolate powder	10	0.08–1.85	1.116 ± 0.832 ^a^	0.280–0.820	0.593 ± 0.183 ^a^	0.230–0.560	0.399 ± 0.107 ^a^	0.120–0.240	0.164 ± 0.037 ^a^	1.460–3.060	2.322 ± 0.489 ^a^
Bitter chocolate	10	0.26–0.52	0.369 ± 0.079 ^b^	0.140–0.420	0.304 ± 0.099 ^b^	0.001–0.029	0.013 ± 0.009 ^b^	0.004–0.0360	0.018 ± 0.011 ^b,c^	0.526–0.875	0.704 ± 0.101 ^c^
Chocolate wafer	10	0.23–0.93	0.414 ± 0.254 ^b^	0.130–0.280	0.207 ± 0.058 ^b,c^	0.032–0.054	0.043 ± 0.008 ^b^	0.004–0.017	0.010 ± 0.005 ^c^	0.421–1.278	0.674 ± 0.274 ^c^

Min–Max: minimum–maximum; SD: standard deviation; ^a, b, c, d^ Mean values carrying different superscripts small letter on the same column are significantly different (*p* < 0.05).

**Table 2:** Aflatoxin adsorption efficiency in PBS buffer.

Matrix	Time	pH	Aflatoxins Conc. (μg/mL)					
			B_1_	B_2_	G_1_	G_2_	Total AFs	Adsorption%
PBS (−Ve control)	2 h	3.0	n.d.	n.d.	n.d.	n.d.	n.d.	0
		6.8	n.d.	n.d.	n.d.	n.d.	n.d.	0
	4 h	3.0	n.d.	n.d.	n.d.	n.d.	n.d.	0
		6.8	n.d.	n.d.	n.d.	n.d.	n.d.	0
PBS + AFs (+Ve control)	2 h	3.0	0.24 ± 0.01 ^j^	0.26 ± 0.01 ^i^	0.24 ± 0.03 ^i^	0.25 ± 0.02 ^j^	0.99 ± 0.01 ^o^	0
		6.8	0.23 ± 0.02 ^j^	0.25 ± 0.01 ^h^	0.26 ± 0.01 ^j^	0.25 ± 0.01 ^j^	0.99 ± 0.02 ^o^	0
	4 h	3.0	0.25 ± 0.01 ^k^	0.24 ± 0.02 ^h^	0.25 ± 0.03 ^i^	0.26 ± 0.01 ^a^	1.00 ± 0.01 ^o^	0
		6.8	0.24 ± 0.02 ^j^	0.26 ± 0.01 ^i^	0.24 ± 0.01 ^i^	0.25 ± 0.01 ^j^	0.99 ± 0.02 ^o^	0
Activated charcoal	2 h	3.0	0.12 ± 0.01 ^i^	0.13 ± 0.02 ^g^	0.12 ± 0.01 ^g^	0.11 ± 0.02 ^h^	0.48 ± 0.01 ^m^	52.0 ± 0.31 ^l^
		6.8	0.11 ± 0.01 ^h^	0.12 ± 0.01 ^f^	0.13 ± 0.02 ^h^	0.12 ± 0.01 ^h^	0.48 ± 0.01 ^m^	52.0 ± 0.70 ^l^
	4 h	3.0	0.11 ± 0.01 ^h^	0.11 ± 0.01 ^f^	0.12 ± 0.01 ^g^	0.11 ± 0.01 ^h^	0.45 ± 0.01 ^l^	55.0 ± 0.42 ^k^
		6.8	0.12 ± 0.01 ^i^	0.11 ± 0.02 ^f^	0.13 ± 0.01 ^g^	0.12 ± 0.02 ^h^	0.48 ± 0.02 ^m^	52.0 ± 0.30 ^l^
*L. rhamnosus*	2 h	3.0	0.13 ± 0.03 ^i^	0.12 ± 0.01 ^f^	0.12 ± 0.02 ^g^	0.11 ± 0.02 ^h^	0.48 ± 0.01 ^m^	52.0 ± 0.11 ^l^
		6.8	0.12 ± 0.01 ^i^	0.12 ± 0.01 ^f^	0.13 ± 0.01 ^h^	0.12 ± 0.01 ^h^	0.49 ± 0.01 ^n^	51.0 ± 0.52 ^m^
	4 h	3.0	0.13 ± 0.01 ^i^	0.11 ± 0.01 ^f^	0.12 ± 0.01 ^a^	0.11 ± 0.01 ^h^	0.47 ± 0.01 ^m^	53.0 ± 0.01 ^l^
		6.8	0.13 ± 0.01 ^i^	0.12 ± 0.01 ^f^	0.11 ± 0.02 ^g^	0.13 ± 0.01 ^i^	0.49 ± 0.02 ^n^	51.0 ± 0.05 ^m^
*S. cerevisiae*	2 h	3.0	0.08 ± 0.01 ^g^	0.07 ± 0.02 ^e^	0.08 ± 0.01 ^f^	0.06 ± 0.01 ^f^	0.29 ± 0.01 ^k^	71.0 ± 0.04 ^j^
		6.8	0.08 ± 0.02 ^a^	0.08 ± 0.01 ^e^	0.06 ± 0.01 ^e^	0.07 ± 0.02 ^f,g^	0.29 ± 0.02 ^k^	71.0 ± 0.14 ^j^
	4 h	3.0	0.07 ± 0.01 ^f^	0.06 ± 0.01 ^d^	0.08 ± 0.02 ^f^	0.06 ± 0.01 ^f^	0.27 ± 0.01 ^j^	73.0 ± 0.11 ^i^
		6.8	0.08 ± 0.01 ^g^	0.07 ± 0.02 ^d^	0.06 ± 0.01 ^e^	0.08 ± 0.02 ^g^	0.29 ± 0.01 ^a^	71.0 ± 0.07 ^j^
A. charcoal *+ L. rhamnosus*	2 h	3.0	0.06 ± 0.01 ^e^	0.05 ± 0.01 ^c^	0.05 ± 0.01 ^d^	0.04 ± 0.01 ^e^	0.20 ± 0.01 ^h^	80.0 ± 0.41 ^g^
		6.8	0.05 ± 0.01 ^d^	0.04 ± 0.01 ^c^	0.06 ± 0.01 ^e^	0.05 ± 0.01 ^e^	0.20 ± 0.02 ^h^	80.0 ± 0.31 ^g^
	4 h	3.0	0.05 ± 0.01 ^d^	0.06 ± 0.01 ^e^	0.05 ± 0.01 ^d^	0.06 ± 0.01 ^f^	0.22 ± 0.01 ^i^	78.0 ± 0.09 ^h^
		6.8	0.04 ± 0.01 ^d^	0.05 ± 0.01 ^d^	0.06 ± 0.01 ^e^	0.05 ± 0.01 ^e^	0.20 ± 0.01 ^h^	80.0 ± 0.14 ^g^
A. charcoal + *S. cerevisiae*	2 h	3.0	0.03 ± 0.01 ^c^	0.02 ± 0.01 ^c^	0.03 ± 0.01 ^c^	0.02 ± 0.01 ^d^	0.10 ± 0.01 ^e^	89.0 ± 0.21 ^d^
		6.8	0.03 ± 0.01 ^c^	0.03 ± 0.01 ^c^	0.02 ± 0.01 ^c^	0.02 ± 0.01 ^d^	0.10 ± 0.01 ^e^	89.0 ± 0.07 ^d^
	4 h	3.0	0.02 ± 0.02 ^b,c^	0.02 ± 0.01 ^c^	0.03 ± 0.03 ^c^	0.02 ± 0.02 ^d^	0.09 ± 0.02 ^e^	91.0 ± 0.22 ^c^
		6.8	0.02 ± 0.0 ^b,c^	0.03 ± 0.01 ^c^	0.02 ± 0.01 ^c^	0.02 ± 0.01 ^d^	0.09 ± 0.01 ^e^	91.0 ± 0.17 ^c^
*L. rhamnosus* + *S. cerevisiae*	2 h	3.0	0.05 ± 0.01 ^d^	0.03 ± 0.01 ^c^	0.04 ± 0.01 ^d^	0.05 ± 0.01 ^e^	0.17 ± 0.01 ^g^	83.0 ± 0.11 ^e,f^
		6.8	0.04 ± 0.01 ^d^	0.04 ± 0.01 ^d^	0.03 ± 0.01 ^c^	0.04 ± 0.01 ^e^	0.15 ± 0.02 ^f^	85.0 ± 0.06 ^e^
	4 h	3.0	0.03 ± 0.01 ^c^	0.05 ± 0.02 ^d^	0.04 ± 0.01 ^d^	0.03 ± 0.01 ^d^	0.15 ± 0.01 ^f^	85.0 ± 0.32 ^e^
		6.8	0.03 ± 0.02 ^c^	0.04 ± 0.01 ^d^	0.05 ± 0.01 ^d^	0.04 ± 0.02 ^e^	0.16 ± 0.01 ^f^	84.0 ± 0.41 ^e^
A. charcoal + *L. rhamnosus* + *S. cerevisiae*	2 h	3.0	0.01 ± 0.01 ^b^	0.01 ± 0.01 ^b^	0.007 ± ±0.02 ^b^	0.005 ± 0.01 ^c^	0.032 ± 0.02 ^d^	96.8 ± 0.08 ^b^
		6.8	0.01 ± 0.01 ^b^	0.006 ± 0.02 ^a^	0.004 ± 0.01 ^a^	0.003 ± 0.01 ^a^	0.023 ± 0.01 ^c^	97.7 ± 0.45 ^a,b^
	4 h	3.0	0.005 ± 0.01 ^a^	0.003 ± 0.01 ^a^	0.005 ± 0.01 ^a^	0.006 ± 0.01 ^b^	0.019 ± 0.01 ^b^	98.1 ± 0.41 ^a^
		6.8	0.003 ± 0.01 ^a^	n.d.	n.d.	n.d.	0.003 ± 0.01 ^a^	99.7 ± 0.13 ^a^

^a, b, c, d, e, f, g, h, i, j, k, l, m, n, o^ Mean values carrying different superscripts small letter on the same column are significantly different (*p* < 0.05). n.d.: not detected. PBS: Phosphate buffered saline; −Ve control: negative control (PBS); +Ve control: positive control + AFs; n.d.: Not detected.

**Table 3:** Aflatoxin adsorption efficiency in supplemented chocolate with tri-mix.

Sample	Time	pH	Aflatoxins Conc. (μg/mL)					
			B_1_	B_2_	G_1_	G_2_	Total AFs	Adsorption%
Chocolate (−Ve control)	2 h	3.0	n.d.	n.d.	n.d.	n.d.	n.d.	0
		6.8	n.d.	n.d.	n.d.	n.d.	n.d.	0
	4 h	3.0	n.d.	n.d.	n.d.	n.d.	n.d.	0
		6.8	n.d.	n.d.	n.d.	n.d.	n.d.	0
Chocolate + AFs (+Ve control)	2 h	3.0	0.24 ± 0.02 ^c^	0.25 ± 0.01 ^c^	0.24 ± 0.01 ^c^	0.25 ± 0.03 ^c^	0.98 ± 0.01 ^c^	0
		6.8	0.24 ± 0.02 ^c^	0.26 ± 0.01 ^d^	0.25 ± 0.02 ^c^	0.24 ± 0.06 ^c^	0.99 ± 0.02 ^c^	0
	4 h	3.0	0.25 ± 0.01 ^c^	0.24 ± 0.02 ^c^	0.25 ± 0.01 ^c^	0.24 ± 0.03 ^c^	0.98 ± 0.01 ^c^	0
		6.8	0.25 ± 0.03 ^c^	0.25 ± 0.01 ^c^	0.24 ± 0.03 ^c^	0.25 ± 0.01 ^c^	0.99 ± 0.01 ^c^	0
Chocolate + AF + tri-mix (A. charcoal + *L. rhamnosus* + *S. cerevisiae*)	2 h	3.0	0.021 ± 0.02 ^b^	0.016 ± 0.01 ^b^	0.005 ± 0.01 ^a^	0.004 ± 0.02 ^b^	0.046 ± 0.02 ^a^	95.40 ± 0.11 ^c^
		6.8	0.020 ± 0.01 ^b^	0.015 ± 0.01 ^b^	0.06 ± 0.02 ^b^	0.003 ± 0.02 ^a^	0.098 ± 0.01 ^b^	90.20 ± 0.24 ^c^
	4 h	3.0	0.017 ± 0.02 ^a,b^	0.015 ± 0.01 ^b^	0.005 ± 0.01 ^a^	0.002 ± 0.03 ^a^	0.039 ± 0.01 ^a^	96.10 ± 0.47 ^a^
		6.8	0.014 ± 0.0 ^a^	0.013 ± 0.01 ^a^	0.004 ± 0.01 ^a^	0.001 ± 0.01 ^a^	0.032 ± 0.01 ^a^	96.80 ± 0.15 ^a^

^a, b, c, d^ Mean values carrying different superscripts small letter on the same column are significantly different (*p* < 0.05). n.d.: not detected. −Ve control: negative control or/chocolate; +Ve control: positive control or/chocolate + AFs; n.d.: Not detected.

**Table 4:** Estimation of cytotoxicity of charcoal and IC_50_ (µg/mL).

Sample	Concentration (µg/mL)	Viability%	Inhibition%
Charcoal	390	18	82
	195	26	74
	97.5	29	71
	48.75	47	53
	24.37	58	42
	12.18	72	28
	6.09	94	6

IC_50_ for charcoal: 45.9 µg/mL.

**Table 5:** Sensory properties of tri-mix-fortified dark chocolate.

Treatment/Group	Sensorial Properties Mean ± SD					
	Color	Odor	Taste	Texture	Appearance	Overall Acceptance
Control	8.4 ± 0.96 ^a^	8.5 ± 0.84 ^a^	8.6 ± 0.84 ^a^	8.6 ± 0.84 ^a^	7.9 ± 1.37 ^a^	8.6 ± 0.84 ^a^
A. charcoal + *L. rhamnosus* + *S. cerevisiae*	8.4 ± 0.94 ^a^	8.1 ± 0.99 ^a^	8.0 ± 1.05 ^b^	8.0 ± 1.05 ^b^	7.8 ± 1.31 ^a^	8.3 ± 0.78 ^a^

^a, b^ Mean values carrying different superscript small letter on the same column are significantly different (*p* < 0.05); tri-mix adsorbents: activated charcoal + *L. rhamnosus* + *S. cerevisiae.*

## Data Availability

Not applicable.
